# Phospholipase D Family Member 4, a Transmembrane Glycoprotein with No Phospholipase D Activity, Expression in Spleen and Early Postnatal Microglia

**DOI:** 10.1371/journal.pone.0013932

**Published:** 2010-11-11

**Authors:** Fumio Yoshikawa, Yoshiko Banno, Yoshinori Otani, Yoshihide Yamaguchi, Yuko Nagakura-Takagi, Noriyuki Morita, Yumi Sato, Chihiro Saruta, Hirozumi Nishibe, Tetsushi Sadakata, Yo Shinoda, Kanehiro Hayashi, Yuriko Mishima, Hiroko Baba, Teiichi Furuichi

**Affiliations:** 1 Laboratory for Molecular Neurogenesis, RIKEN Brain Science Institute, Wako, Saitama, Japan; 2 Department of Cell Signaling, Gifu University Graduate School of Medicine, Gifu, Gifu, Japan; 3 Department of Molecular Neurobiology, Tokyo University of Pharmacy and Life Sciences, Hachioji, Tokyo, Japan; 4 Yasuda Women's University, Hiroshima, Hiroshima, Japan; 5 JST, CREST, Kawaguchi, Saitama, Japan; 6 Saitama University Graduate School of Science and Engineering, Saitama, Saitama, Japan; 7 Hiroshima University Graduate School of Biomedical Sciences, Hiroshima, Hiroshima, Japan; Universidade Federal do Rio de Janeiro, Brazil

## Abstract

**Background:**

Phospholipase D (PLD) catalyzes conversion of phosphatidylcholine into choline and phosphatidic acid, leading to a variety of intracellular signal transduction events. Two classical PLDs, PLD1 and PLD2, contain phosphatidylinositide-binding PX and PH domains and two conserved His-x-Lys-(x)_4_-Asp (HKD) motifs, which are critical for PLD activity. PLD4 officially belongs to the PLD family, because it possesses two HKD motifs. However, it lacks PX and PH domains and has a putative transmembrane domain instead. Nevertheless, little is known regarding expression, structure, and function of PLD4.

**Methodology/Principal Findings:**

PLD4 was analyzed in terms of expression, structure, and function. Expression was analyzed in developing mouse brains and non-neuronal tissues using microarray, *in situ* hybridization, immunohistochemistry, and immunocytochemistry. Structure was evaluated using bioinformatics analysis of protein domains, biochemical analyses of transmembrane property, and enzymatic deglycosylation. PLD activity was examined by choline release and transphosphatidylation assays. Results demonstrated low to modest, but characteristic, PLD4 mRNA expression in a subset of cells preferentially localized around white matter regions, including the corpus callosum and cerebellar white matter, during the first postnatal week. These PLD4 mRNA-expressing cells were identified as Iba1-positive microglia. In non-neuronal tissues, PLD4 mRNA expression was widespread, but predominantly distributed in the spleen. Intense PLD4 expression was detected around the marginal zone of the splenic red pulp, and splenic PLD4 protein recovered from subcellular membrane fractions was highly N-glycosylated. PLD4 was heterologously expressed in cell lines and localized in the endoplasmic reticulum and Golgi apparatus. Moreover, heterologously expressed PLD4 proteins did not exhibit PLD enzymatic activity.

**Conclusions/Significance:**

Results showed that PLD4 is a non-PLD, HKD motif-carrying, transmembrane glycoprotein localized in the endoplasmic reticulum and Golgi apparatus. The spatiotemporally restricted expression patterns suggested that PLD4 might play a role in common function(s) among microglia during early postnatal brain development and splenic marginal zone cells.

## Introduction

Phospholipase D (PLD) is an important phospholipid signaling enzyme, which catalyzes conversion of phosphatidylcholine (PC) into choline and phosphatidic acid (PA) following activation by diverse cellular signaling molecules, such as hormones, growth factors, and neurotransmitters [1], [2], [3]. PA is, in turn, converted into two second messengers, diacylglycerol (a protein kinase C activator) and lysophosphatidic acid (LPA) (ligand for G-protein-coupled LPA receptors; see [4]), which are involved in regulation of membrane and vesicle trafficking, cell proliferation, migration, and actin cytoskeleton dynamics [1], [5], [6], [7]. PA has been implicated in diverse cellular functions due to activity with target proteins [8]. In the brain, PLD also plays a pivotal role in neuronal proliferation and survival, neurite outgrowth, and neurodegeneration [9], [10], [11]. There are two distinct genes in mammals, PLD1 and PLD2 [12], [13], [14], [15]. In the N-terminal region, PLD1 and PLD2 proteins contain Phox homology (PX) and pleckstrin homology (PH) domains, both of which are involved in membrane targeting of PLD [16], [17], [18], [19], which leads to membrane localization and activation of PLD. In the C-terminal region, PLD1 and PLD2 contain two conserved His-x-Lys-x-x-x-x-Asp sequences (where x is any amino acid) and are designated HKD motifs; these are essential for PLD enzymatic activity [20]. In addition to the enzymatically, well-characterized, classical PLDs (PLD1 and PLD2), a bioinformatic homology search for the HKD motif identified a superfamily of PLD-like HKD motif-containing proteins. This superfamily consists of eight protein classes, including bacterial cardiolipin synthase (CLS), phosphatidylserine synthase (PSS) and endonuclease (Nuc), and *Vaccinia* virus p37K and K4L [21]. Human Hu-K4 [22], [23] and mouse SAM9 [24], which are now officially designated “PLD family member 3 (PLD3)”, lack PX and PH domains and exhibit no PLD activity, despite two HKD motifs [23], [24]. There are three additional PLD family members: “PLD family member 4 (PLD4)”, “PLD family member 5 (PLD5)”, and “PLD family member 6 (PLD6)”. However, little is known about these non-classical PLDs, which have a putative TM domain instead of PX and PH domains.

The present study identified a developmentally regulated transcript, PLD4, after searching the cerebellar development transcriptome database (CDT-DB) for characteristic spatiotemporal gene expression patterns during mouse cerebellar development [25]. The basic properties of PLD4 have not been reported, but these findings identified unique features of PLD4 in the amino acid sequence, as well as functions and expression, compared with other members of the PLD superfamily.

## Results

### Amino acid sequence comparison of mouse PLD4 with PLD superfamily members

**Figure 1:**
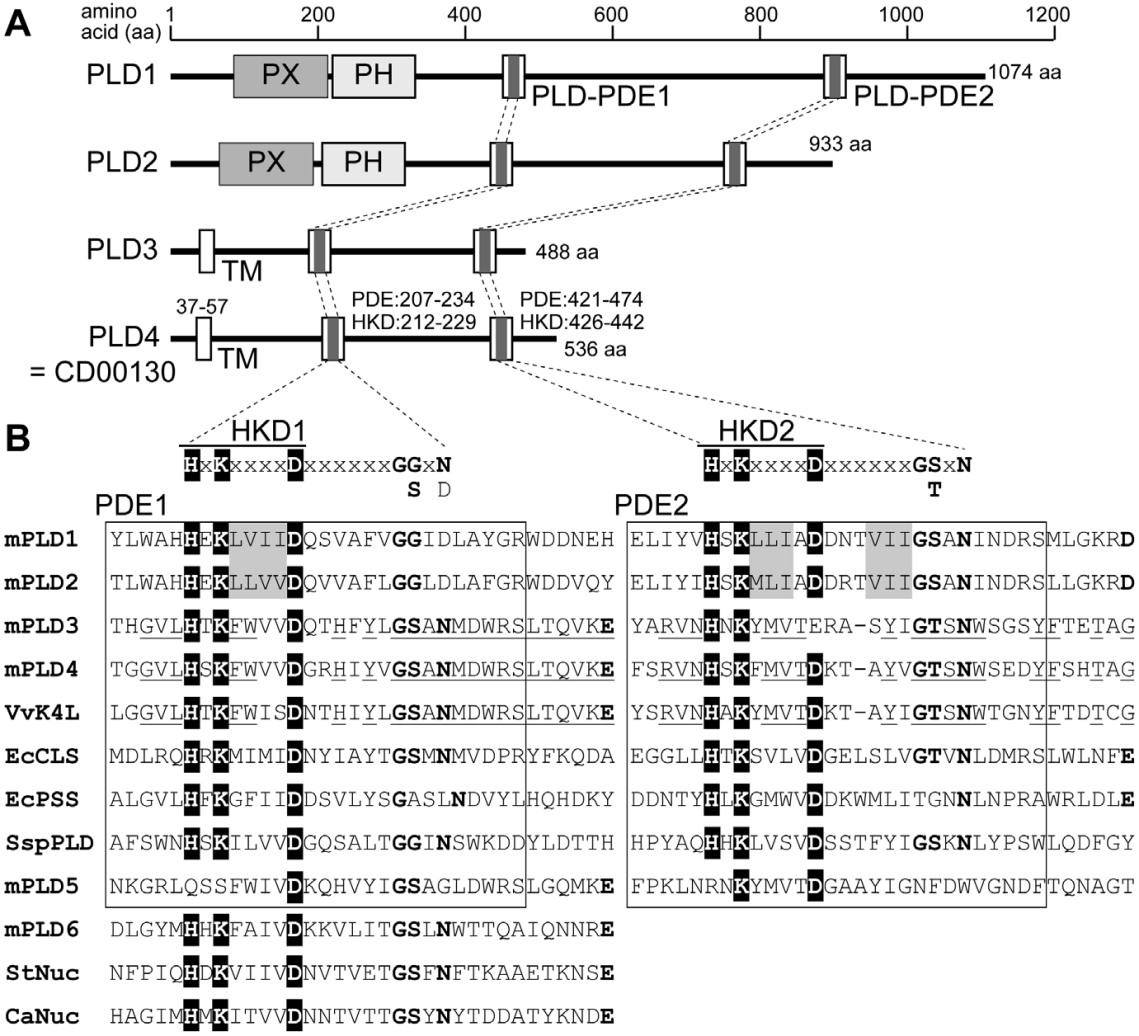
Structural comparisons of PLD superfamily members. A, protein structures and domains of mouse PLD family members PLD1, PLD2, PLD3, PLD4 (CDT-DB ID = CD00130), PLD5, and PLD6. Top line represents amino acid (aa) position. PX, Phox homology domain; PH, pleckstrin homology domain; PLD-PDE1 and PLD-PDE2, PLD-phosphodiesterase domain 1 and 2, respectively; TM, transmembrane domain. B, amino-acid sequence alignment of the PLD-PDE domains containing conserved HKD motifs of PLD superfamily members. mPLD1–6, PLD1–6 of mice; VvK4L, K4L protein of *Vaccinia* virus; EcCLS, cardiolipin synthase of *Escherichia coli*; EcPSS, phosphatidylserine syntase of *E. coli*; SspPLD, PLD of *Streptomyces sp.* strain PMF; StNuc, nuclease of *Salmonella typhimurium* IncN plasmid; CaNuc, nuclease of *Clostoridium acetobtylicum*. The amino-acid sequences of two PDE domains (PDE1 and PDE2) containing the conserved His-x-Lys-x-x-x-x-D (where x is any amino acid) motif (HKD1–2, motif 1 and motif 2, respectively) are aligned. PDE1 and PDE2 domain sequences are surrounded by a frame. Conserved His (H), Lys (K), and Asp (D) residues are highlighted black. mPLD6, StNuc, and CaNuc contain only one HKD motif and the HKD-containing sequences are aligned with PDE1-HKD1 domains from other members. Conserved amino acid residues from the superfamily are in bold. Conserved hydrophobic patches in mPLD1 and mPLD2 are highlighted grey. Conserved amino acid residues of mPLD3, mPLD4, and VvK4L are underlined.

By analyzing gene expression profiles of CDT-DB during postnatal mouse cerebellar development, a developmentally regulated transcript was identified (CDT-DB ID number CD00130; DDBJ/GenBank/EMBL accession number BP426385). Subsequent cloning and sequencing analysis identified this mRNA transcript as PLD4 (Fig. S1). PLD4 is an official member of the PLD family of enzymes. This family is composed of six members (PLD1–6), all of which contain a PLD-phosphodiesterase (PDE) domain (PLD-PDE) consisting of a conserved HKD motif (Fig. 1A). In the C-terminal region, PLD family members (with the exception of PLD6) possess two PDE domains, PLD-PDE1 and PLD-PDE2, which contain HKD – HKD1 and HKD2 – respectively (Fig. 1B). HKD motifs are critical for PLD activity [20] and are involved in forming the active site of phosphate binding, as shown by crystal structural analyses of *Salmonella* Nuc [26] and *Streptomyces* PLD [27] (shown as StNuc and SspPLD in Fig. 1B, respectively). In the N-terminal region, classical PLDs (PLD1 and PLD2) with PLD activity contain PX and PH domains; these proteins will hereafter be referred to as PXPH-type PLDs [12], [13], [15], [17], [20], [28]. PLD1 and PLD2 share 45% identity in overall amino acid sequences. However, the non-classical PLD (PLD3–6) contains a putative transmembrane (TM) domain in place of PX and PH domains. In the present study, PLD3 and PLD4 were analyzed, which we will refer to as TM-type PLDs, because PLD5 contains less conserved sequences in the HKD motifs, and PLD6 has only a single HKD motif. The TM-PLDs PLD3 and PLD4 exhibited only 28% identity between the amino-acid sequences. However, PLD3 and PLD4 exhibited the greatest homology in the PLD-PDE1 and PLD-PDE2 domains, which are also highly homologous with the *Vaccinia* virus K4L PLD-like protein (66.7% and 53.8% identity, respectively), compared with PXPH-PLDs (14.8% and 15.4% identity, respectively) and other superfamily members (Fig. 1B). In addition to the lack of PX and PH domains, TM-PLDs likely possess altered amino-acid sequences in the critical domains that correspond to the PXPH-PLD phosphate-binding structure.

### PLD4 mRNA expression is prominent during the first postnatal week in the mouse cerebellum and exhibits greatest expression in the mouse spleen

**Figure 2:**
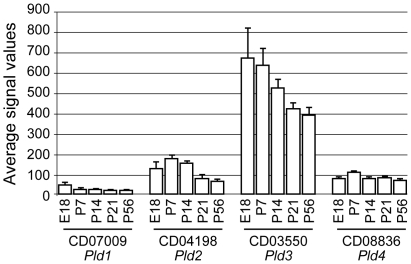
Temporal profiles of mouse PLD1–4 mRNA expression during the postnatal period. Expression profiles of PLD1–4 (*Pld1*, CD07009; *Pld2*, CD04198; *Pld3*, CD03550; *Pld4*, CD08836 in the CDT-DB) mRNAs in mouse cerebella at E18, P7, P14, P21, and P56 were analyzed using GeneChip microarrays (Affymetrix MG 430 2.0 Array). Average values of normalized hybridization signals are presented. Error bars show standard error of the mean (SEM) from four independent experiments.

Temporal expression analysis by GeneChip microarrays revealed low levels of PLD4 mRNA expression during postnatal development of the mouse cerebellum (Fig. 2). This was confirmed by semi-quantitative RT-PCR (Fig. S2A). PLD4 mRNA expression gradually increased after birth, peaked at P7, and was significantly reduced thereafter (Fig. S2A,B). These results suggested a role for PLD4 in early cerebellar development. Within the PLD family, another TM-PLD – PLD3 – exhibited the highest mRNA expression levels during the postnatal stage; expression peaked around the first postnatal week and then gradually declined with age (Fig. 2A). Among the PXPH-PLDs, expression of PLD1 mRNA was low and gradually decreased after birth, whereas PLD2 expression was greater and peaked at P7 before decreasing thereafter (Fig. 2A).

**Figure 3:**
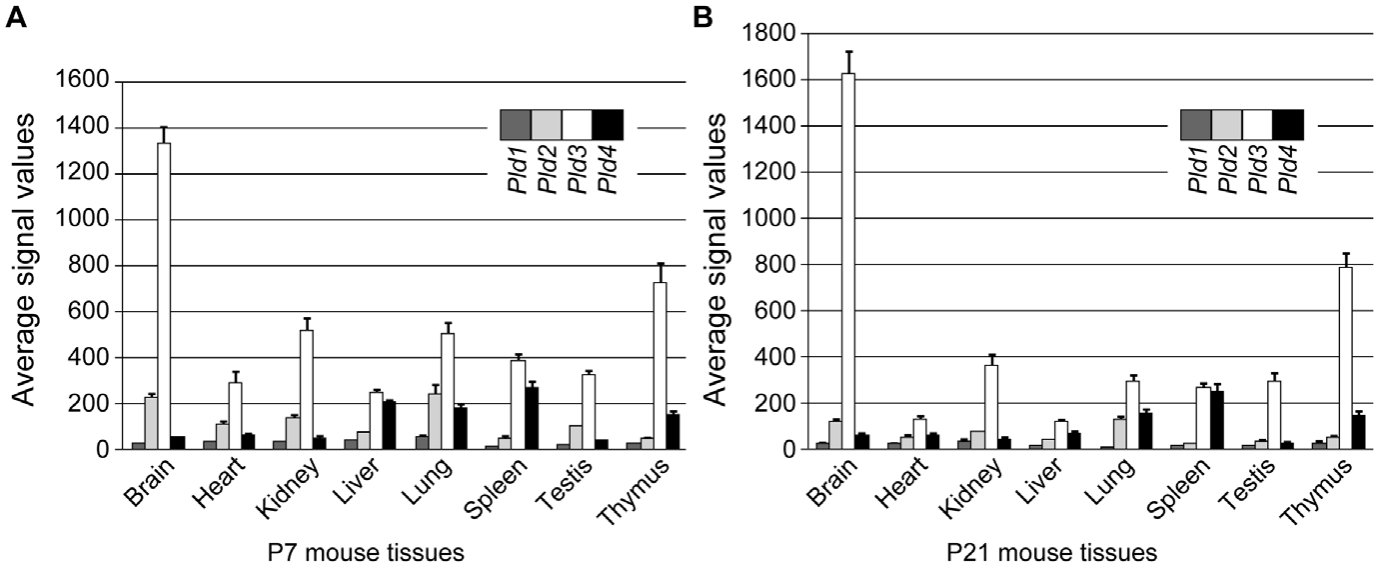
Tissue distribution of mouse PLD1–4 mRNA expression. Expression profiles of PLD1–4 (*Pld1*, CD07009; *Pld2*, CD04198; *Pld3*, CD03550; *Pld4*, CD08836 in the CDT-DB) mRNAs in the brain, heart, kidney, liver, lung, spleen, testis, and thymus tissues of C57BL6/J mice at P7 (A) and P21 (B) were analyzed using GeneChip microarrays (Affymetrix MG 430A 2.0 Array). Average values of normalized hybridization signals are indicated (mean ± SEM, n = 3).

Expression patterns of PLD4 mRNA in eight different tissues (brain, heart, kidney, liver, lung, spleen, testis, and thymus) of P7 and P21 mice were determined with GeneChip microarrays (Fig. 3). Among the tissues tested, signal values for PLD4 mRNA were very low in the brain at P7 and P21 compared with PLD3 mRNA. The highest level was observed in the spleen, and moderate levels were detected in the liver, lung, and thymus at P7, as well as in the lung and thymus at P21. In contrast, PLD3 mRNA expression was greatest in the brain, followed by the thymus. However, it should be noted that in the spleen (at P7 and P21) and liver (at P7), PLD4 and PLD3 levels were comparable, and these levels were greater than PLD1 and PLD2.

### PLD4 mRNA expression is restricted to a subset of cells located within the white matter region during the early postnatal stage

**Figure 4:**
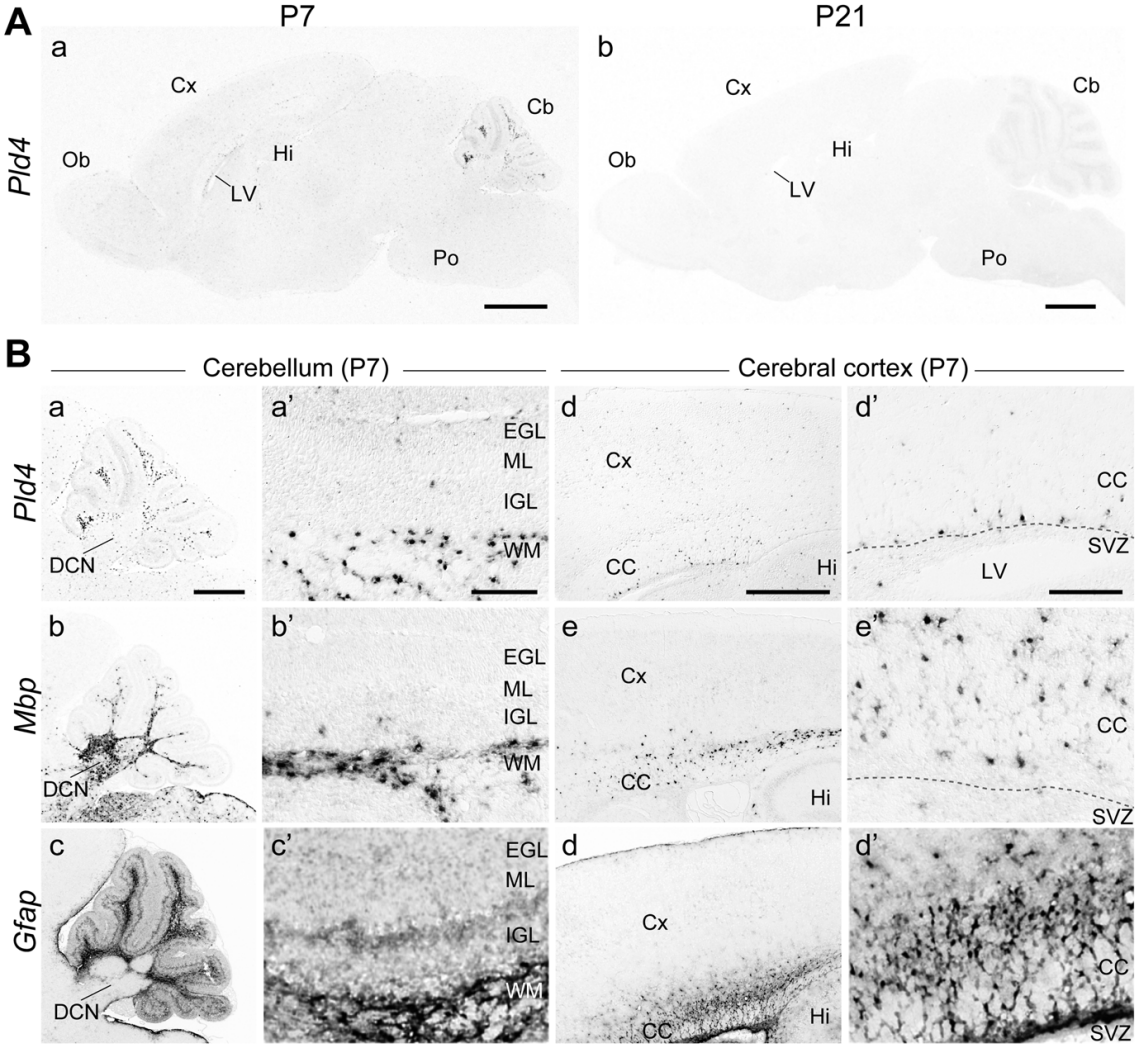
Spatial cellular profiles of PLD4 mRNA expression in mouse brains as determined by *in situ* hybridization. A, distribution of PLD4 mRNA in sagittal sections of mouse brains at P7 (a) and P21 (b). Scale bars = 1 mm. B, images of cerebellum (panels a–c and a'–c') and cerebral cortex (panels d–f and d'–f') expressing PLD4, MBP, and GFAP mRNAs at P7. Regions of mRNA signals in panels a–f are magnified in panels a'–f', respectively. Cb, cerebellum; CC, corpus callosum; Cx, cerebral cortex; DCN, deep cerebellar nuclei; EGL, external granular layer; Hi, hippocampus; IGL, internal granular layer; LV, lateral ventricle; ML, molecular layer; Ob, olfactory bulb; Po, pons; SVZ, subventricular zone; WM, white matter. Scale bars = 500 µm in a; 100 µm in a', d and d'.

Cellular expression of PLD4 mRNA in P7 and P21 mouse brain sections was analyzed by *in situ* hybridization (ISH) (Fig. 4). At P7, PLD4 mRNA signals were weak (Fig. 4A-a), but modestly localized, in a subset of cells located in the white matter of cerebellar lobules, but not in the deep cerebellar nuclei (DCN) region (Fig. 4B-a and a'). These signals were also observed in a subset of cells located in a subregion of the corpus callosum (CC) and near the subventricular zone (SVZ) (Fig. 4B-d and d'). PLD4 mRNA expression was distinguishable from myelin basic protein (MBP) mRNA expression in oligodendrocytes clustered within the cerebellar white matter (Fig. 4B-b and b') and CC (Fig. 4B-e and e'). PLD4 mRNA expression was also distinguishable from glial fibrillary acidic protein (GFAP) mRNA expression in astrocytes (Fig. 4B-c, c', d and d'), neural stem cells, and radial glia in the SVZ (Fig. 4-d and d'). At P21, detectable signals for PLD4 mRNA were not identified in any brain regions using these methods (Fig. 4A-b). These results demonstrated that PLD4 mRNA is expressed in cell type(s) preferentially localized around white matter and throughout brain regions during the early postnatal period.

### PLD4 is expressed in white matter microglial cells in early postnatal mouse brains and is predominantly localized in cells within the red pulp of mouse spleens

**Figure 5:**
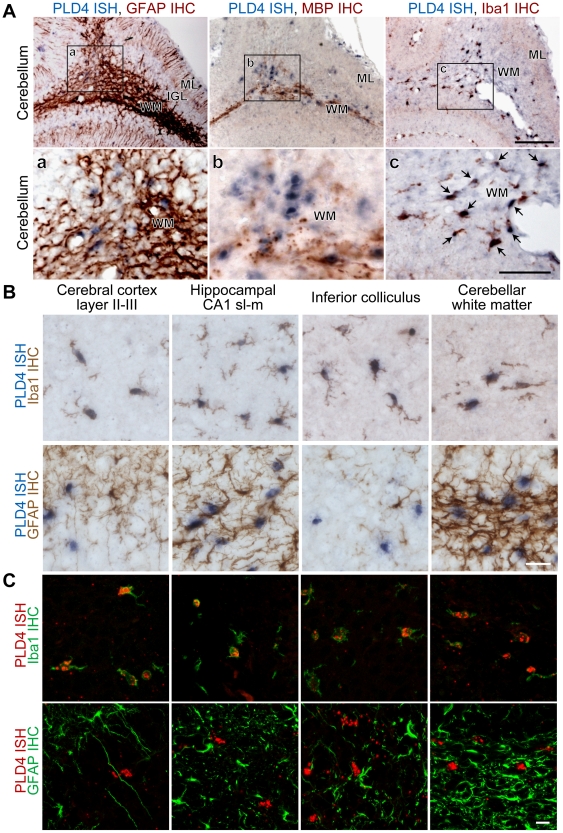
Mouse PLD4 mRNA expression is localized in white matter microglia of early postnatal brains. A, Double labeling analysis of mouse cerebellar sections using *in situ* hybridization (ISH) of PLD4 mRNA and immunohistochemistry (IHC) with glial cell marker proteins. PLD4 mRNA (dark blue color) and protein (brown color) for GFAP (astrocyte marker) (left panel), MBP (oligodendrocyte marker) (middle panel), or Iba1 (microglia marker) (right panel) are simultaneously detected around the white matter region of the mouse cerebellum at P7. Panels a, b, and c show enlarged images of the frame indicated in the top panels. B and C, double labeling of layer II–III in the cerebral cortex, the stratum-lacunosum-moleculare (sl-m) of the hippocampal CA1 region, inferior colliculus, and cerebellar white matter by ISH for PLD4 mRNA and IHC (green) for Iba1 or GFAP. Conventional NBT/BCIP chromagen detection (dark blue) in B and HNPP/Fast Red fluorescent detection (red) in C were used. ISH signals for PLD4 mRNA are merged with IHC signals for Iba1 protein in these brain regions. WM, white matter; IGL, internal granular layer; ML, molecular layer. Scale bars = 100 µm and 50 µm in top and bottom of panel A, respectively; 20 µm in B and 10 µm in C.

To identify cell types that express PLD4 mRNA, double labeling was performed by ISH and immunohistochemistry (IHC), respectively, on cerebellar sections of P7 mice (Fig. 5). ISH signals of PLD4 mRNA were localized to the cerebellar white matter region and did not merge with IHC signals for GFAP or MBP (Fig. 5A). This was consistent with data obtained from ISH, as presented in Fig. 4. Interestingly, PLD4 ISH signals in the cerebellar white matter closely matched IHC signals for Iba1 (a microglial marker) [29] (as shown in the rightmost panels of Fig. 5A–C). Some PLD4 mRNA-expressing cells were also scattered within layer II–III of the cerebral cortex, the stratum-lacunosum-moleculare (sl-m) of the hippocampal CA1, and the inferior colliculus; these cells also expressed Iba1 protein, but not GFAP (Fig. 5B and C). These results demonstrated that PLD4 mRNA was expressed in microglial cells in early postnatal mouse brains.

**Figure 6:**
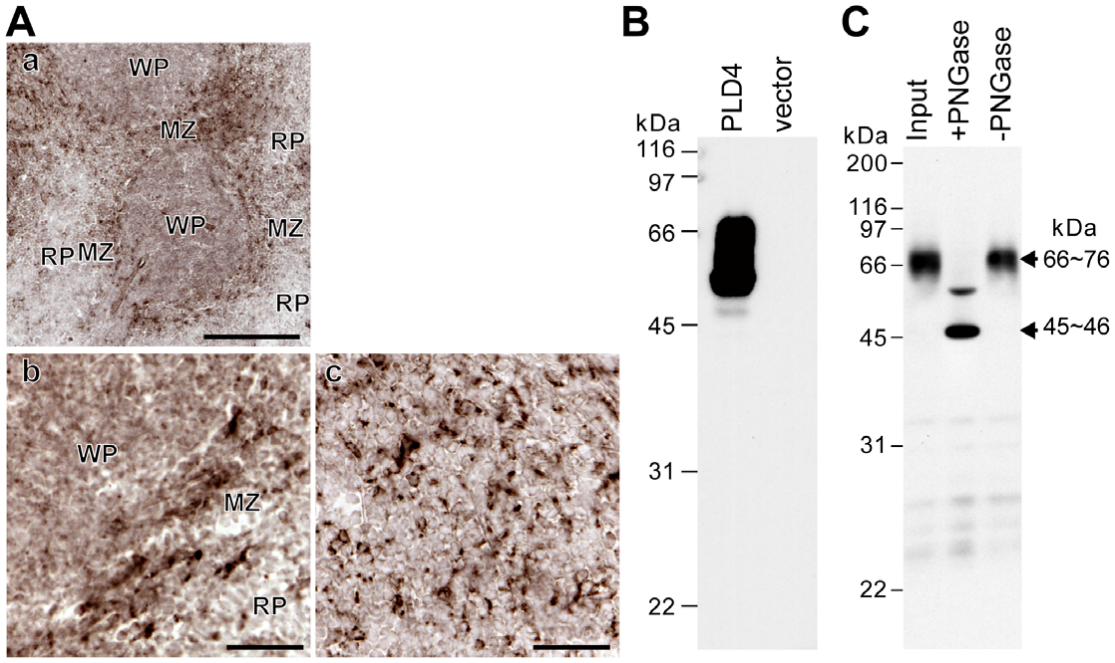
PLD4 protein expression and glycosylation. A, Mouse PLD4 protein expression is observed in cells within the red pulp, and largely around the marginal zone, of adult spleens. Immunohistochemical labeling of adult mouse spleen sections with anti-PLD4 antibody. PLD4 immunolabels are largely detected in splenic marginal zone cells within the red pulp. MZ, marginal zone; RP, red pulp; WP, white pulp. Scale bars = 500 µm. B, Western blot analysis of PLD4 proteins exogenously expressed in COS7 cells using an anti-PLD4 antibody. PLD4 and vector represent protein lysates of cells transfected with pcDNA3-PLD4 and pcDNA3 vector alone, respectively. C, Deglycosylation of endogenous PLD4 proteins in the mouse spleen membrane fraction by N-glycosidase-F (PNGase) digestion. Input, protein samples before treatment; +PNGase and -PNGase, treated with and without PNGase, respectively. The major immunoreactive bands of approximately 45∼46 kDa and 66∼76 kDa after treatment with and without PNGase, respectively, are indicated with arrows. Left, sizes in kDa of molecular weight markers.

The mouse spleen expressed the highest levels of PLD4 mRNA among the different tissues tested, as shown in Figure 3. Although it was difficult to detect reliable PLD4 expression in mouse brains using these conventional methods, PLD4 expression was detected within the red pulp of the mouse spleen and was largely localized in cells around the marginal zone (Fig. 6A).

### PLD4 is a transmembrane glycoprotein located in intracellular organelles

Mouse PLD4 protein, deduced from the cDNA sequence, comprises 503 amino acid residues (calculated molecular mass, 56,154 Da), which contain a putative TM domain (amino acid position [aa] 37–57) (Fig. 1A). However, western blot analysis indicated that protein lysates of COS7 cells transfected with the pcDNA-PLD4 expression vector exhibited a strongly immunoreactive broad band between 50 and 70 kDa (Fig. 6B). Bioinformatics analysis of the predicted PLD4 amino-acid sequence suggested the presence of eight consensus sites of Asn-linked glycosylation (aa 89, 148, 169, 247, 279, 415, 425, and 442) in the C-terminal region, which was downstream of the TM domain (Fig. S1). Therefore, to determine the possibility of glycosylation, PLD4 proteins from mouse spleen were analyzed by enzymatic deglycosylation (because of difficulty with analyzing trace amounts of endogenous PLD4 protein expressed in the cerebellum). In the membrane fraction, but not the soluble fraction, there was strong immunoreactivity for PLD4 at approximately 70 kDa (Fig. 6C). Upon peptide:N-glycosidase (PNGase) F treatment, the major immunoreactive band was shortened to approximately 46 kDa (Fig. 6C). Site-specific mutagenesis of eight Asn-linked glycan consensus sites (Asn to Gln mutations) suggested that PLD4 was exogenously expressed in HEK293 cells and contained large N-glycosylated moieties at multiple consensus sites (Fig. S5B). These results demonstrated that PLD4 is a highly N-glycosylated transmembrane protein.

**Figure 7:**
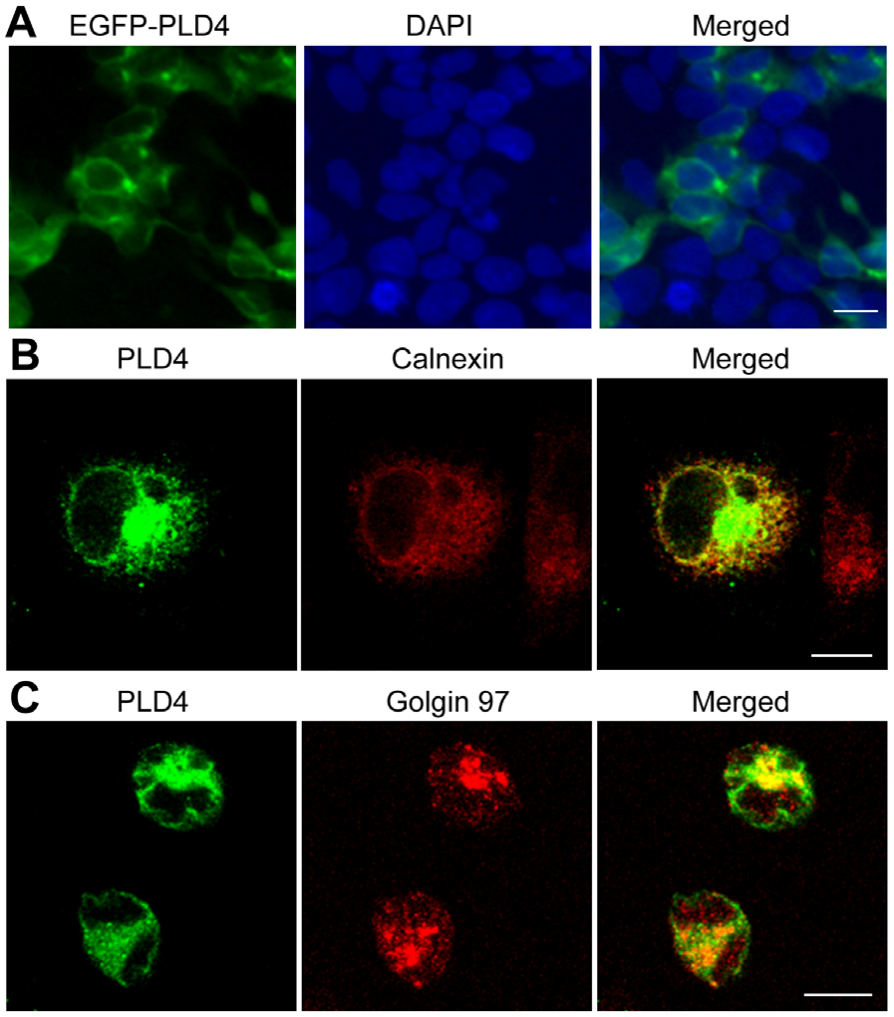
Intracellular localization of PLD4 protein exogenously expressed in cultured cell lines. A, EGFP fluorescent imaging (green) and DAPI nuclear staining (blue) of HEK293 cells transfected with pEGFP-PLD4. PLD4 is localized in a meshwork-like structure and around the periphery of nuclei. B and C, immunocytochemical localization of PLD4 protein (green) and either calnexin, an ER marker (red) (B) or golgin 97, a *trans*-Golgi marker (red) (C), in HeLa cells transfected with pcDNA3-PLD4. PLD4 is colocalized with calnexin in the ER and with golgin 97 in the Golgi complex. Scale bars = 10 µm.

To examine intracellular localization of PLD4, the EGFP-fused PLD4 construct was transfected into four different cell lines (HEK293, COS7, MDCK, and HeLa), and cellular images of EGFP fluorescence were analyzed. EGFP fluorescence formed a meshwork-like structure in the peripheral region of the nucleus in HEK293 cells (Fig. 7A). Similar meshwork-like localization patterns were also observed in COS7 and MDCK cells (data not shown). Double immunostaining for PLD4 and subcellular markers was performed in HeLa cells transfected with pcDNA3-PLD4 (Fig. 7B, C). PLD4 immunoreactivity was co-localized with calnexin, an endoplasmic reticulum (ER) resident protein (Fig. 7B), and PLD4 expression also formed a clustered pattern with golgin 97, a *trans*-Golgi network protein (Fig. 7C). These results suggested that PLD4 protein was localized in organelle membranes, including the ER and Golgi complex.

### PLD4 does not exhibit authentic PLD enzymatic activity

**Figure 8:**
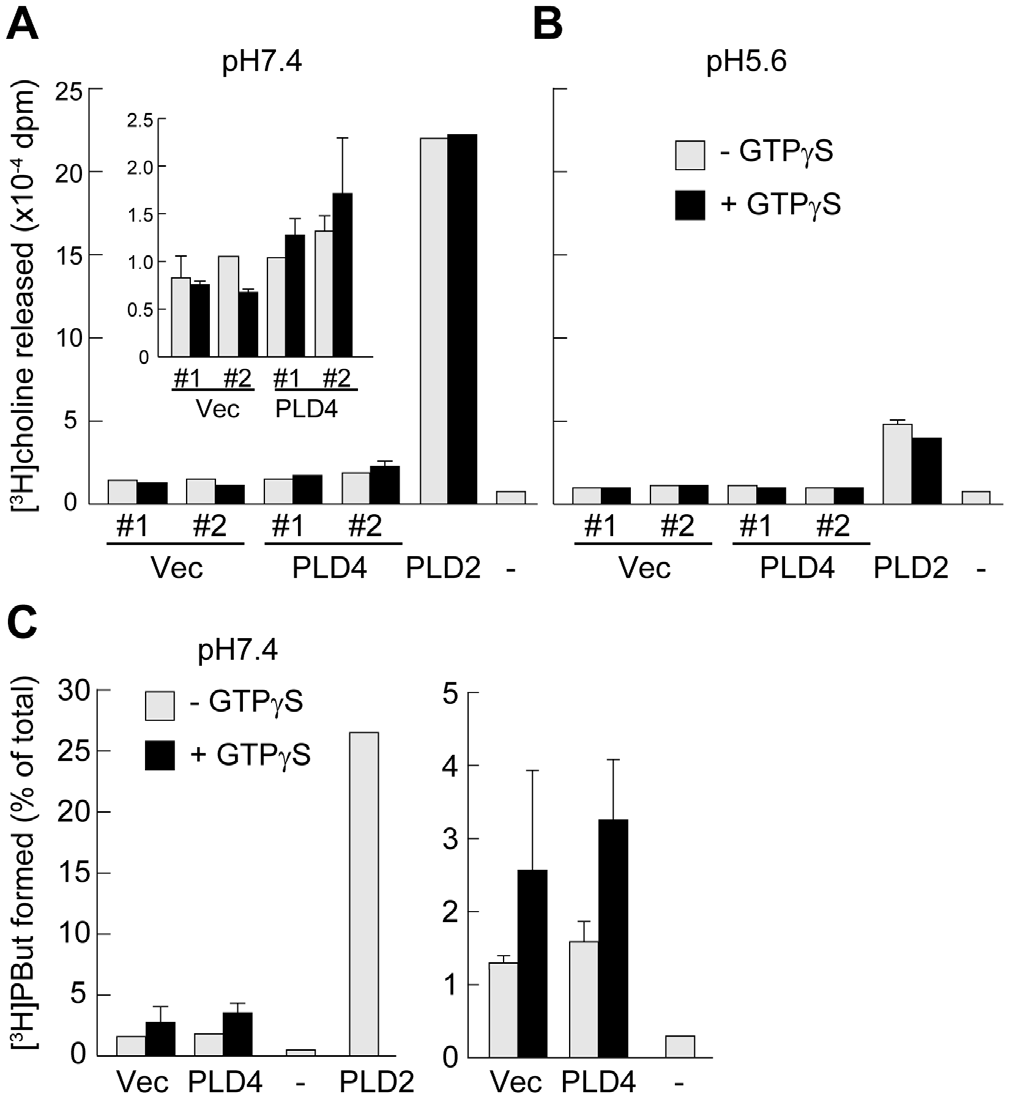
PLD activity assay of PLD4 expression in HEK293 cells. A–B, [^3^H]choline release activity in HEK293 cells transfected with PLD4, PLD2, or pcDNA3 vector alone (Vec) or non-transfected cells (−), in the presence or absence of GTPγS at pH 7.4 (A) or pH 5.2 (B). The inset in A shows the same data for vector (Vec)-transfected and PLD4-transfected cells on a different scale. Data from two independent experiments (#1 and #2, n = 3) are shown for PLD4 and Vec. C, Transphosphatidylation activity of HEK293 cells transfected with PLD4, PLD2, or vector alone (Vec), or non-transfected cells (−), in the presence or absence of GTPγS. The right graph shows the same data for Vec, PLD4, and – on a different scale.

As shown in Figure 1B, comparative alignment of the HKD motif amino acid sequence, which is essential for PLD enzymatic activity, demonstrated the presence of several differences between TM-PLDs and the PXPH-PLDs (see Discussion). Therefore, PLD activity was examined in mouse PLD4 proteins, which were exogenously expressed in HEK293 cells, by analyzing choline release and transphosphatidylation activities in the presence or absence of GTPγS (mammalian PLD1 is activated by G proteins [2]). Protein lysates of cells transfected with PLD2 (positive control) exhibited high [^3^H]choline release activity at pH 7.4 (Fig. 8A) and retained a weak, but significant, level of activity at pH 5.2 (Fig. 8B), regardless of the presence or absence of GTPγS. However, cell lysates transfected with PLD4 did not exhibit significant PLD activity, such as cells transfected with vector alone. Finally, transphosphatidylation activity of PLD was analyzed using 1-butanol as the nucleophile to generate [^3^H]phosphatidylbutanol (PBut). Protein lysates of PLD4-expressing cells did not form a significant amount of [^3^H]PBut, compared with PLD2-expressing cells (Fig. 8C). These results suggested that PLD4 does not exhibit authentic PLD activity, although deglycosylated PLD4 might possess PLD activity.

## Discussion

The present study identified the characteristic mRNA expression profile of PLD4 during mouse brain development. Results demonstrated that PLD4 is a transmembrane protein containing Asn-linked glycans and it is intracellularly localized to organelle membranes, such as the ER and Golgi. PLD4 expression in HEK293 cells did not exhibit authentic PLD enzymatic activity. In addition, results demonstrated that PLD4 was not a classical PLD (PLD1 or PLD2), and PLD4 might play a role as an organellar membrane-associated protein in a subset of Iba1-positive microglial cells spatiotemporally enriched in the white matter of early postnatal mouse brains, as well as in splenic marginal zone cells.

Previous studies reported that PLD1 and PLD2 mRNAs [13], [14], [15], [30], as well as PLD3 mRNA [23], [24], are highly expressed in various brain regions and in many non-neuronal tissues. In contrast, temporal and cellular expression patterns of PLD4 mRNA are fairly restricted to developing mouse brains and various tissues. This expression was particularly marked in a small cell population of the white matter layer in cerebellar lobules, as well as in the CC near the SVZ, during the first postnatal week. Some expressing cells were also scattered in the cerebral cortical layer, the sl-m of the hippocampal CA1, and the inferior colliculus. These PLD4 mRNA-positive cells were identified as Iba1-positive microglia, which likely corresponded to a subset of microglia that transiently appeared in white matter of developing brains [31], [32], [33], [34]. It was recently demonstrated that PLD4 expression in microglial cells exhibits an extracellular stimulus-dependent response (Ohtani, Yamaguchi, and Baba, manuscript in preparation). Among the non-neuronal tissues tested, PLD4 mRNA levels were greatest in the spleen, and intense PLD4 expression was detected around the splenic marginal zone, where macrophages are located [35].

PLD4 contains eight Asn-linked glycosylation consensus sites within the C-terminal region (Fig. S1). The present study demonstrated that exogenously expressed PLD4 was an integral membrane protein carrying the Asn-linked glycan moiety; PLD4 was localized in a meshwork-like ER structure and in the Golgi (possibly in the nuclear membrane as well), although it was difficult to localize endogenous PLD4 in neuronal cells using the present methods. These results suggested that PLD4 as a type-2 transmembrane glycoprotein within organellar membranes. It contained a short N-terminus region that faces the cytoplasm and a long C-terminal putative catalytic domain containing HKD motifs, which reside within the lumen. It has been shown that human PLD3 (also known as Hu-K4) protein is exogenously expressed in COS7 cells and is also a glycosylated type-2 transmembrane protein in the ER [23].

Considering transmembrane topology, TM-PLDs might exert activity against substrates or targets within the organelle (luminal side), but not on the cytoplasmic side. However, membrane-associated PXPH-PLDs catalyze cytoplasm-facing substrates in the plasma membrane and intracellular compartments, including the ER, endosomes, and secretory granules (Cockcroft, 2001). The present results demonstrated that protein lysates of PLD4-transfected HEK293 cells exhibited no obvious PLD activity, even at pH 5.2, which was similar to acidity conditions within secretory granules (∼pH 5.5) or the Golgi lumen (∼pH 6.2 or 7.4, which is identical to near neutral pH of the cytoplasm and ER lumen) [36], [37]. However, it remains to be determined whether PLD4 exhibits enzymatic activity and whether the deglycosylated form of PLD4 possesses PLD activity.

Two HKD motifs (HKD1 and HKD2) are conserved in the PLD superfamily, with the exception of PLD6 and Nuc; these HKD motifs are critical for PLD activity in PXPH-PLDs [20]. Crystal structural analyses of *Salmonella* Nuc (StNuc) [26] and *Streptomyces* PLD (SspPLD) [27] (Fig. 1B) have revealed that HKD motifs and the flanking sequences form a phosphate-binding site. Taking into account previous results of the structure-function relationship between HKD motifs and PLD activity, the following structural features of TM-PLDs, which were distinguishable from PXPH-PLDs, will be discussed. First, three hydrophobic amino-acid patches (highlighted with grey in Fig. 1B), which are located within the phosphate-binding tertiary structure, according to SspPLD data [27], are important for catalytic activity, as well as for the inter-domain association between HKD1 and HKD2 in the rat PLD1 [28]. In contrast, TM-PLDs (and also K4L) contain the aromatic amino acids Phe (F), Trp (W), and Tyr (Y) in corresponding sequences (Fig. 1B). Second, Gly-Gly (GG) and Gly-Ser (GS) motifs, which are located seven residues downstream of HKD1 and HKD2, respectively, are well-conserved among the PXPH-PLDs. A mutation of N-terminal GG to GS increases transphosphatidylation activity in *Streptoverticillium cinnamoneum* PLD (ScPLD) [38]. Interestingly, TM-PLDs and K4L contain GS at the N-terminal end, although the present data demonstrated that PLD4 exhibited no significant transphosphatidylation activity (Fig. 8C). In the C-terminal GS motif, it has been shown that the Ser residue contributes to thermal enzymatic stability in ScPLD [38], and a mutation that changes GS to GT results in reduced enzymatic activity to 39% of human PLD1 levels [20]. The C-terminal motif in TM-PLDs and K4L is GT, instead of GS (Fig. 1B), which might be related to the loss of PLD activity in these family members. Third, tertiary structure analysis of StNuc demonstrates that the Glu (E) residue 29 residues downstream of the first HKD2 amino acid is involved in forming the active site (Struckey and Dixon, 1999) (Fig. 1B). Family members with enzymatic activity, including PXPH-PLDs, *E. coli* CLS, and PSS, contain either Glu (E) or Asp (D) downstream of HKD2, although this residue is a Tyr (Y) in SspPLD (Fig. 1B). However, in TM-PLDs and K4L, Glu (E) is located opposite of 29 residues downstream of HKD1, but not HKD2. The structural differences in the catalytic active site between TM-PLDs and PXPH-PLDs might be associated with enzymatic properties, such as substrate specificity.

In conclusion, these results suggested that PLD4 is a HKD motif-containing protein family member that lacks authentic PLD activity, which is most likely due to structural alterations in PX/PH domains and HKD motifs critical for enzymatic activity. Moreover, the unique spatiotemporal expression profiles of PLD4 suggest a role in cell function(s), which are common between early postnatal white matter microglia and splenic marginal zone cells, including macrophages.

## Materials and Methods

### Animals

Mice (ICR and C57BL/6J) and rabbits (New Zealand White) were purchased from Nihon SLC (Hamamatsu, Japan). All animal studies were conducted according to recommendations and protocols approved by the Animal Care and Use Committee of RIKEN (approval number: H21-2-244(4)) and the Tokyo University of Pharmacy and Life Sciences (approval number: Y09–15). Animals were housed in an environment with 12:12-hour light/dark cycle (daytime 8:00–20:00), controlled temperature (23±2°C) and humidity (55±10%), and *ad libitum* access to food and water.

### Cloning and plasmid construction of PLD cDNAs

The first clone (clone ID number CD00130 see CDT-DB [www.cdtdb.brain.riken.jp]; DDBJ/GenBank/EMBL accession number BP426385) was isolated by fluorescent differential display analysis of the mouse cerebellar transcriptome during postnatal developmental stages, as previously described [25]. Full-length PLD4 cDNAs were cloned by screening full-length cDNA libraries. The cDNA libraries were generated from poly(A)^+^ mRNAs isolated from mouse cerebella at P7 using the oligo-capping method for sequence homology to clone CD00130 as previously described [39]. The full-length cDNA sequence was determined using the dideoxynucleotide termination method with a DNA sequencer (ABI 3730xl, Applied Biosystems, Foster City, CA). Mouse PLD2 cDNA was similarly cloned by RT-PCR and was used following sequence verification. PLD4 and PLD2 cDNA were cloned into the mammalian expression vector pcDNA3 (Invitrogen, Rockville, MD). For enzymatic activity analysis, the mouse PLD2 expression vector pcGN-mPLD2 [14], which was kindly provided by Dr. M. Frohman, was utilized.

### Reverse-transcription polymerase chain reaction (RT-PCR)

cDNAs were prepared from total RNAs obtained from mouse cerebella (ICR) at embryonic day (E)18 and at postnatal days (P)0, P3, P7, P12, P21, and P56. PCRs were performed with 25 or 28 cycles in a thermocycler (GeneAmp PCR system 9700, ABI) as previously described [25], [39]. Forward and reverse primers for amplification of each gene were: PLD4, 5′-GACTGGAGTTCCCACTATGCTAT-3′ and 5′-AGGTGGCAGGGTTTTATTGTGGCT-3′, respectively; glyceraldehyde-3-phosphate dehydrogenase (GAPDH), 5′-GCCATCAACGACCCCTTCATTGACCTC-3′ and 5′-GCCATGTAGGCCATGAGGTCCACCAC-3′, respectively. GAPDH was utilized as a constant signal standard.

### GeneChip analysis

RNA preparation and GeneChip reactions were performed as previously described [25]. Gene expression profiles during cerebellar development of C57BL/6J mice (E18, P7, P14, P21, and P56) were analyzed using the GeneChip system Affymetrix Mouse Genome 430 2.0 Array (GE Healthcare UK, Buckinghamshire, England), which included probes for approximately 39,000 transcripts. Tissue distribution of gene expression was analyzed in eight different mouse tissues (brain, thymus, lung, heart, liver, spleen, kidney, and testis) (C57BL/6J) at P7 or P21 using the Affymetrix Mouse Genome 430A 2.0 Array (GE Healthcare), which included probes for approximately 15,000 transcripts. Data were normalized and analyzed using GeneSpring GX software (Agilent Technologies, Santa Clara, CA) as previously described [25].

### *In situ* hybridization

Brain *in situ* hybridization (ISH) was performed as previously described [25], [39]. The template sequences were used to generate riboprobes as follows: PLD4 (GenBank NM_178911), 153-bp fragment amplified with 5′-primer AGGGCTGAGGGTTGATCC (nucleotide position [nt] 1613–1630) and 3′-primer CTGCCGGAAGCTGTTTGT (nt 1748–1765); MBP (NM_001025259), 162-bp fragment amplified with 5′-primer TCGCCACAATAACGTGAG (nt 1774–1791) and 3′-primer TGCTTCTGTCCAGCCATACT (nt 1976–1935); GFAP (NM_010277), 400-bp fragment amplified with 5′-primer CACGAACGAGTCCCTAGA (nt 908–907) and 3′-primer TCACATCACCACGTCCTT (nt 1290–1307). Sections (6- or 8-µm thick) from 4% paraformaldehyde-fixed, paraffin-embedded, P7 and P21 mouse brains (ICR) were deparaffinized and treated with a proteinase K solution (10 ng/µl in PBS; Invitrogen, Carlsbad, CA, USA) at room temperature for 15 min. Following acetylation, the sections were incubated in hybridization buffer containing 1 µg/ml digoxigenin-labeled riboprobes at 60°C overnight in a humidified chamber. In addition to PLD4 riboprobes produced from PCR amplicon as a template, hybridization in Fig. 5B–C was performed using 2 µg/ml of alkaline-hydrolyzed PLD4 riboprobe which was prepared from 1.5-kb full-length PLD4 cDNA as a template by *in vitro* transcription. Hybridized sections were washed by successive immersion in 2× SSC, 50% formamide (60°C for 20 min, twice), TNE (1 mM EDTA, 0.5 M NaCl, 10 mM Tris-HCl, pH 8.0, at 37°C for 10 min), TNE containing 20 ng/µl RNase A (37°C for 30 min), 2× SSC (room temperature for 10 min, twice), and 0.2× SSC (60°C for 30 min, twice). Hybridization signals were detected using a digoxigenin detection kit (Roche Diagnostics) and examined using microscopes (SMZ-U and Eclipse E800; Nikon, Tokyo, Japan) equipped with a cooled CCD camera (Spot; Diagnostic Instruments, Sterling Heights, MI). Digital images were processed using Adobe Photoshop 6.0 software.

### Antibodies

The antigenic synthetic peptide of mouse PLD4 consisted of C-terminal 16 amino acid residues (from amino acid 488 to 503, YAMDLDRQVPSQDCVW) with no homology to the C-termini of the other mouse PLD family members (Fig. S3). Female, New Zealand, white rabbits (Nihon-SLC) were immunized by subcutaneous injection of peptide conjugated to keyhole limpet hemocyanin (catalog number [Cat.] H7017, Sigma-Aldrich, St. Louis, MO). The resultant PLD4 antibody (FD130-1#4) was purified by affinity chromatography on an antigenic peptide-coupled SulfoLink column (SulfoLink Immobilization Kit for Peptides, product #44999, Pierce, Rockford, IL) and utilized at a concentration of 1 µg/ml. Specificity of the anti-PLD4 antibody was verified by a pre-absorption test with the antigenic peptide (Fig. S4C), as well as by specific recognition of expressed recombinant proteins and similarity in developmental and tissue distribution patterns between immunoreactive protein (Fig. S4B) and mRNA (Fig. S2). Other primary antibodies used were goat polyclonal anti-calnexin antibody (1∶1000) (Cat. sc-6465, Santa Cruz Biotechnology, Santa Cruz, CA), mouse monoclonal anti-golgin-97 antibody (1∶1000) (A-21270, Invitrogen), rabbit polyclonal anti-ionized calcium-binding adaptor molecule 1 (Iba1) antibody (1∶400) (Cat. 019-19741, Wako Pure Chemical Industries, Osaka, Japan), rabbit polyclonal anti-glial fibrillary acidic protein (GFAP) antibody (1∶10) (Cat. N1506, Dako, Glostrup, Denmark), and rat monoclonal anti-myelin basic protein (MBP) antibody (1∶100) (Cat. MAD386, Chemicon/Millipore, Temecula, CA). The secondary antibody for western blot analyses was horseradish peroxidase (HRP)-conjugated anti-rabbit IgG (H+L) (1∶2000) (Cat. NA9340, GE Healthcare UK Ltd.). The secondary antibodies applied to immunocytochemical analyses were Alexa Fluor 488-conjugated anti-rabbit IgG (1∶2000) (Cat. A11029, Invitrogen), Alexa Fluor 594-conjugated anti-goat IgG (1∶2000) (Cat. A11058, Invitrogen), and Alexa Fluor 594-conjugated anti-mouse IgG (1∶2000) (Cat. A11005, Invitrogen). The secondary antibodies used for immunohistochemical analyses were biotinylated anti-rabbit IgG (Cat. PK-4001) and anti-rat IgG (Cat. PK -4004) (1∶250) from the Vectastain ABC kit (Vector Laboratories, Burlingame, CA, USA).

### Subcellular fractionation

Spleens were dissected from mice (ICR) under deep diethyl ether anesthesia and were homogenized in an ice-cold Teflon-glass potter homogenizer containing 9 vol (w/v%) homogenizing buffer (0.25 M sucrose, 5 mM Tris-HCl, pH 7.4) and protease inhibitor cocktail (1 mM phenylmethylsulfonyl fluoride, 10 µM pepstatin A, 10 µM leupeptin) as previously described [39]. The homogenates were centrifuged at 1000×*g* for 10 min at 4°C to obtain precipitate fraction 1 (ppt1). The supernatants were then centrifuged at 105,000×*g* for 1 h at 4°C to obtain precipitate fractions 2+3 (ppt2+3), and the cytosolic fraction was obtained from the supernatant. Ppt1 and ppt2+3 were re-suspended in suspension buffer (20 mM Tris-HCl, pH 7.4, 150 mM NaCl, 1 mM DTT, and protease inhibitor cocktail), and protein concentrations were measured using a BCA protein assay kit (Pierce, Rockford, IL).

### Western blot analysis

Protein lysates were separated by SDS-polyacrylamide gel electrophoresis and were electro-blotted onto nitrocellulose filter membranes (Hybond-ECL, Cat. RPN2020D, GE Healthcare UK) as previously described [39]. The blots were incubated at room temperature for 1 h in blocking buffer consisting of 5% (w/v) skim milk (Snow Brand, Sapporo, Japan) and 1× PBS containing 0.1% (v/v) Tween 20 (PBS-T). Blots were then incubated with primary antibody in PBS-T for 1 h, followed by HRP-conjugated secondary antibody for 1 h. After washing with PBS-T, the bound antibody was detected using ECL Plus western blotting detection reagent (Cat. RPN2106, GE Healthcare UK Ltd.), and images captured on X-ray film (MXJB Film, Cat. 864-8651, Eastman Kodak, Rochester, NY).

### Enzymatic deglycosylation

The deglycosylation reaction was performed as previously described [39]. Briefly, protein samples were pre-incubated in 1% SDS, 144 mM 2-mercaptoethanol, and protease inhibitor cocktail at 37°C for 30 min. Asn-linked glycans were removed by treatment with 30 U/ml *N*-glycosidase F (PNGase, from *Flavobacterium meningosepticum*) (Roche Diagnostics, Mannheim, Germany) in 20 mM sodium phosphate buffer (pH 7.2), 0.5% NP40, 20 mM EDTA, 1 mM 2-mercaptoethanol, and protease inhibitor cocktail overnight at 37°C.

### Cell culture and transfection

HeLa, COS7, MDCK, and HEK293 cell lines were obtained from the Cell Bank of RIKEN Bioresource Center (Tsukuba, Japan) and were cultured in DMEM in a humidified atmosphere with 5% CO_2_ at 37°C. Transfection was performed using Lipofectamine 2000 reagent (Invitrogen) as previously described [39].

### Immunocytochemistry

Cultured cells were rinsed with PBS, fixed with 4% PFA/PBS at room temperature for 15 min, rinsed three times in PBS, and then permeabilized in 0.2% Triton X-100 in PBS for 5 min. After blocking with 5% normal donkey serum (Cat. D9663, Sigma-Aldrich) in PBS at room temperature for 1 h, the cells were incubated with anti-PLD4 antibody at 4°C overnight, rinsed in PBS, and then incubated with Alexa Fluor-conjugated secondary antibody in PBS at room temperature for 1 h before being rinsed again in PBS. Immunostained sections were mounted using Vectashield mounting medium (Vector Laboratories, Burlingame, CA) and were examined using a confocal laser microscope (LSM 510 META; Carl Zeiss, Oberkochen, Germany). Digital images were processed using Adobe Photoshop 6.0 software.

### Immunohistochemistry and double labeling by *in situ* hybridization and immunohistochemistry

Immunohistochemistry (IHC) of frozen sections prepared from mouse spleens was performed as previously described [39]. C57BL/6J mice (Nihon SLC) were anesthetized with diethyl ether and transcardially perfused with PBS, followed by 4% PFA/PBS. The spleens were dissected, post-fixed in 4% PFA at 4°C for 1 h, and cryoprotected by immersion in 20% sucrose in PBS overnight at 4°C. After embedding in Tissue-Tek OCT compound (Sakura Finetechnical, Tokyo, Japan), the spleens were frozen in dry-ice powder and cut into 10–16-µm thick sagittal sections at −18°C using a cryostat (CM1850; Leica Microsystems, Wetzlar, Germany). The sections were then air-dried for 1 h, rinsed three times in PBS, and incubated with methanol at −20°C for 20 min, followed by three washes with PBS at 4°C for 10 min each. After blocking with 10% normal donkey serum (Cat. D9663, Sigma-Aldrich) in 0.2% Triton X-100 and PBS, the sections were incubated with anti-PLD4 antibody at 4°C overnight. Sections were then rinsed in 0.2% Triton X-100 in PBS and allowed to react with biotin-conjugated anti-rabbit IgG (1∶1000) in 5% normal serum, 0.2% Triton X-100, and PBS at room temperature for 1 h. The immunostaining was detected using a Vectastain ABC kit (0.05% diaminobenzidine [DAB]/0.01% H_2_O_2_). Images were captured using a microscope (Nikon Eclipse800, or Olympus BX51) equipped with a cooled CCD camera (SPOT; Diagnostic Instruments, Sterling Heights, MI, or ProgRes C14; Jepoptik, Munich, Germany). For double staining of mRNAs and proteins in mouse brain paraffin sections (P7 old, 6-µm thick), following ISH, IHC was performed, as shown in Fig. 5A, 5B and 5C. In Fig. 5A, sections subjected to ISH with the PLD4 PCR amplicon probes were boiled in citrate buffer (pH 6.0) for 1 min in a microwave oven for heat-induced antigen retrieval. The sections were blocked for 1 hr in 10 mM PBS/0.3% Triton X-100/10% goat serum (PBS-TGS), and then incubated overnight at 4°C with primary antibodies diluted in PBS-TGS. After rinsing in PBS, the sections were incubated with ABC complex (Vectastain ABC kit) for 30 min at RT, and immunoreactions were visualized using H_2_O_2_ in 3,3′-diaminobentidine/50 mM Tris buffer for 10 min at RT. Images were captured using a light microscope (Axio Scope Imaging System; Carl Zeiss, Oberkochen, Germany). In Fig. 5B, following NBT/BCIP staining of ISH with 2 µg/ml of alkaline-hydrolyzed full-length PLD4 riboprobe, sections were incubated with either anti-Iba1 (1∶250) or anti-GFAP antibody (1∶5) at room temperature for 1 hr, followed by biotinylated anti-rabbit IgG at room temperature for 1 hr, and Vectastain ABC kit (0.5 mg/ml DAB/0.01% H_2_O_2_). For double fluorescent by ISH and IHC staining (Fig. 5C), the ISH reaction was first performed using alkaline-hydrolyzed full-length PLD4 riboprobe as described above. Subsequently, the sections were incubated with alkaline phosphatase-labeled anti-DIG antibody (1∶1000) together with either anti-Iba1 or anti-GFAP antibody at 4°C overnight, followed byh Alexa 488-labeled anti-rabbit IgG (1∶1000) at room temperature for 1 hr, and HNPP Fluorescent Detection set (Roche #11 758 888 011). Fluorescent digital images were acquired using a confocal laser-scanning microscope LSM510 (Carl Zeiss). Digital images were processed using Adobe Photoshop software.

### Assay of phospholipase D activity

PLD activity was determined by measuring the generation of ^3^H-labeled choline from [choline-methyl-^3^H]dipalmitoyl-PC (NEN Life Science Products, Boston, MA) (phosphohydrolase activity) and ^3^H-labeled phosphatidyl butanol (PBut) (transphosphatidylation activity) as previously described [40], [41]. Briefly, the HEK293 cells were transfected with either pcDNA3-PLD4 or pcDNA3-PLD2, and were then suspended in ice-cold lysis buffer [20 mM HEPES, pH 7.4, 1 mM EGTA, 1 mM MgCl_2_, 1 mM dithiothreitol, 1 µM phenylmethylsulfonyl fluoride, 20 µg/ml (L-3-*trans*-carboxyoxirane-2-carbonyl)-L-leucyl-agmatine, E-64] and lysed by sonication. Lysates were used for the PLD activity assay following removal of unbroken cells by centrifugation at 1,000×*g* for 5 min. Twenty microliters of lipid vesicles, which contained phoshpatidylethanolamine (PE), phosphatidylinositol 4,5-bisphosphate (PIP_2_), and PC at a molar ratio of 16∶1.4∶1 with [choline-methyl-^3^H]dipalmitoyl-PC (total 4×10^5^ cpm/assay), was added to 100 µl mixture containing a PLD source, 50 mM HEPES-NaOH (pH 7.4 or pH 5.6), 3 mM EGTA, 80 mM KCl, 2.5 mM MgCl_2_, and 2 mM CaCl_2_. The reaction was performed at 37°C for 1 h in the presence or absence of 5 µM GTPγS. Radioactivity of [^3^H]choline, which was released from [choline-methyl-^3^H]dipalmitoyl-PC, was measured.

To assess PLD transphosphatidylation, mixed lipid vesicles (PE/PIP_2_/PC, 16∶1.4∶1 µM) containing [2-palmitoyl-9,10-^3^H]dipalmitoyl-PC (NEN Life Science Products) to yield 4×10^5^ cpm/assay were prepared. A total of 20 µl lipid vesicle was added to 10 µl cell lysate in a total volume of 120 µl containing 50 mM HEPES-NaOH (pH 7.4), 3 mM EGTA, 80 mM KCl, 2.5 mM MgCl_2_, 1.2 mM CaCl_2_, and 0.3% 1-butanol. The reaction was incubated at 37°C for 1 h in the presence or absence of 5 µM GTPγS. After extraction of cellular lipids, [^3^H]PBut was separated by TLC on silica gel LK6D plates (Whatman, Maidstone, UK) using a solvent system of upper-phase ethyl acetate/2,2,4-trimethylpentane/acetic acid/water (13∶2∶3∶10 by volume). The amount of [^3^H]PBut formed was expressed as a percentage of total radioactivity recovered from the TLC plate.

### Bioinformatics analysis

Amino acid sequence data were obtained from NCBI. Protein domain predictions were referred from Protein Knowledgebase UniProtKB. The PLD4 mRNA expression in mouse brains and non-neuronal tissues were analyzed by using transcriptome database CDT-DB.

## Supporting Information

Figure S1 Amino acid sequence and putative protein domains of mouse PLD4. The transmembrane (TM, solid horizontal bar), two PLD-PDE domains (PDE1 and PDE2, rectangles), two HKD motifs (HKD1 and HKD2, grey horizontal bars), and eight Asn-linked glycosylation consensus sites (Asn-89, 148, 169, 247, 279, 415, 425 and 442 with asterisks) are indicated. Asn-397 is marked with an open circle. The Asn residues are mutated to Qln, as shown in Figure S5. Conserved amino acids in the HKD motifs are shown in bold.
(0.46 MB TIF)

Figure S2 Developmental time series expression patterns of PLD4 mRNA during postnatal development of the mouse cerebellum. A, Semi-quantitative RT-PCR analyses at E18, P0, P3, P7, P12, P15, P21, and P56. Two representative results of PLD4 mRNA expression at cycle number 25 and 28, and GAPDH (control) at cycle 25. B, Relative values (%) of PLD4 GeneChip data are shown in A (peak value at P7 is defined as 100%). GeneChip data shown in Figure 2, which shows peak expression of PLD4 mRNA at P7, is verified by these results.
(0.33 MB TIF)

Figure S3 C-terminal amino acid sequences of six mouse PLD family members and the antigen sequence used for generation of the anti-PLD4 antibody. The C-terminal amino acid sequences of six mouse PLD family members (PLD1∼6) are aligned. There is no homology between these C-terminal sequences, except for significant homology between PLD1 and PLD2 (colons indicate identical amino acids). The C-terminal 16 amino acids (highlighted black) of PLD4 were used as the antigenic peptide to generate anti-mouse PLD4 antibody.
(0.18 MB TIF)

Figure S4 Immunodetection of PLD4 protein by anti-PLD4 antibody. A and B, Subcellular protein fractions (ppt1, ppt2+3, and sup3) prepared from mouse cerebellum (at P1, P7, P14, and P21) and spleen (at P7, 7 week [w], and 17w) were immunoblotted with anti-PLD4 antibody. By employing this conventional method (see Materials and Methods), the antibody specifically reacted with a broad 66∼76-kDa band in the ppt2+3 spleen fraction (B), whereas detected only a trace amount of PLD4 in the cerebellum (A). PLD4 protein levels between spleen and cerebellum are consistent with PLD4 mRNA levels indicated by GeneChip analyses shown in Fig. 3. C, Antigen absorption assay of anti-PLD4 antibody. The ppt2+3 protein fractions prepared from the mouse spleen at 7w were immunoblotted with anti-PLD4 antibody in the presence or absence of antigenic peptide (7 µg/ml) at 4°C overnight. The antigen blocked immunodetection, indicating specificity of the anti-PLD4 antibody to the antigenic amino acid sequence.
(0.45 MB TIF)

Figure S5 Analyses of glycosylated PLD4 by enzymatic deglycosylation and site-specific mutagenesis. A, Deglycosylation of endogenous PLD4 in the mouse spleen with N-glycosidase-F (PNGase), as shown in Figure 6B. Non-specific bands are indicated by asterisks. B, Site-specific mutagenesis of eight Asn-linked glycan consensus sites in PLD4. Consensus Asn residues (N) at 89, 148, 169, 247, 279, 415, 425, and 442 were individually substituted by Gln (Q). Mutated PLD4, except for N425 and N442Q, exhibits slightly faster mobility than wild-type PLD4 (WT). However, the non-consensus Asn-397 was mutated to Q (N397Q) as a control and exhibits similar mobility as WT. These results suggest that exogenously expressed PLD4 has large and complex glycan moieties and at least six consensus Asn residues at 89, 148, 169, 247, 279, and 415. However, the *in vivo* glycosylation sites of PLD4 protein remain to be shown.
(0.27 MB TIF)
