# Supplementary figures and images for: Phospholipase D Family Member 4, a Transmembrane Glycoprotein with No Phospholipase D Activity, Expression in Spleen and Early Postnatal Microglia
Source: PLoS One. 2010 Nov 11;5(11):e13932. doi: 10.1371/journal.pone.0013932 (PMC2978679; doi:10.1371/journal.pone.0013932)

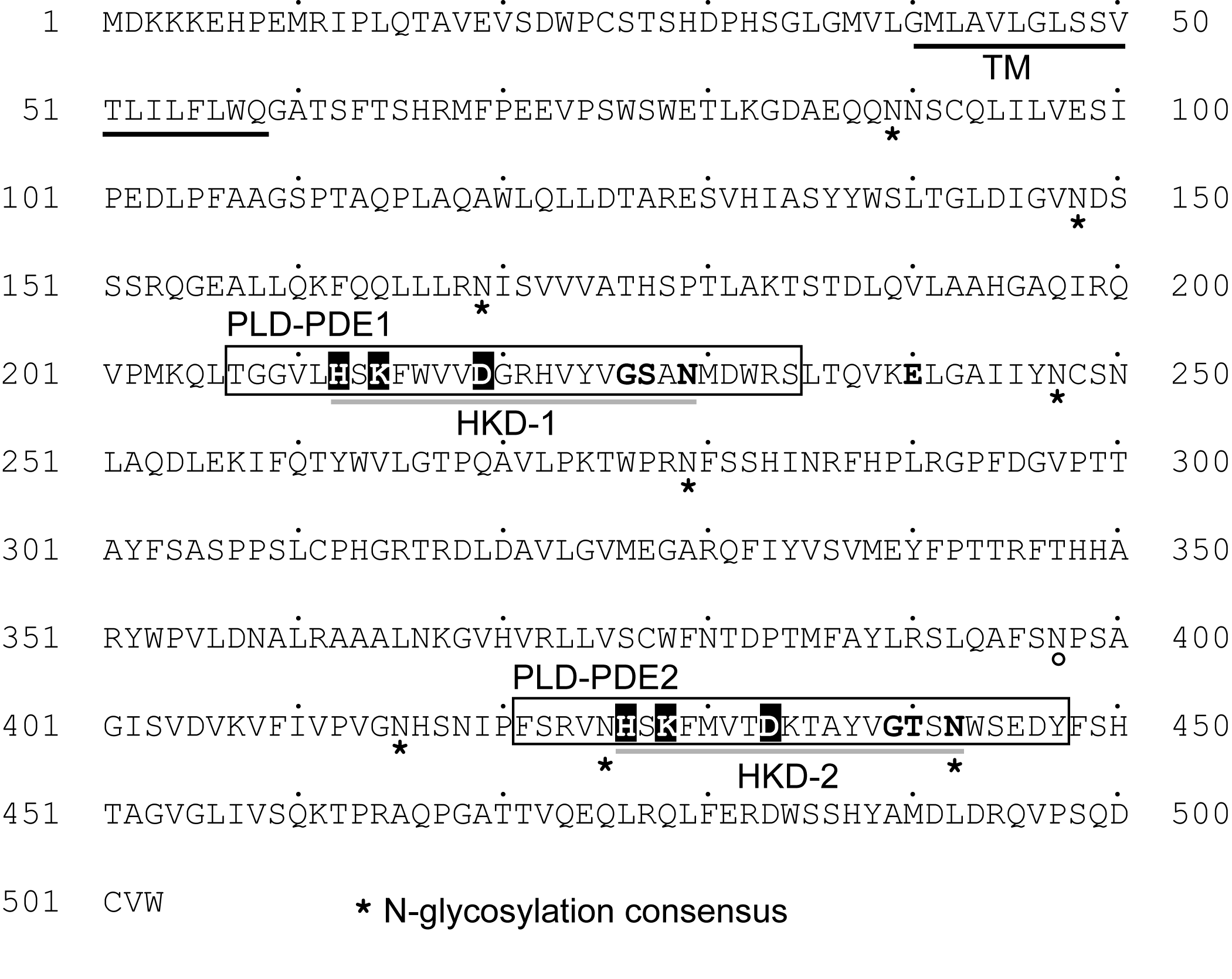

Supplement: Figure S1 — Amino acid sequence and putative protein domains of mouse PLD4. The transmembrane (TM, solid horizontal bar), two PLD-PDE domains (PDE1 and PDE2, rectangles), two HKD motifs (HKD1 and HKD2, grey horizontal bars), and eight Asn-linked glycosylation consensus sites (Asn-89, 148, 169, 247, 279, 415, 425 and 442 with asterisks) are indicated. Asn-397 is marked with an open circle. The Asn residues are mutated to Qln, as shown in Figure S5. Conserved amino acids in the HKD motifs are shown in bold. (0.46 MB TIF) [file pone.0013932.s001.tif]

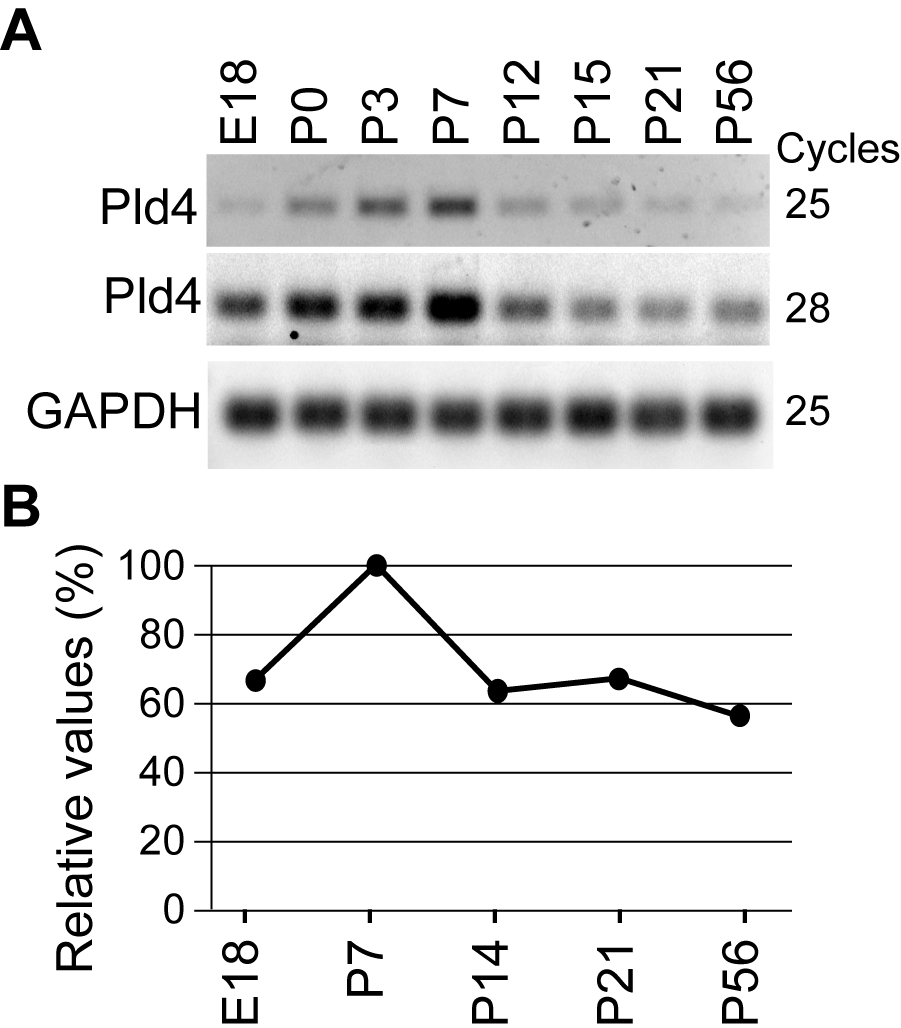

Supplement: Figure S2 — Developmental time series expression patterns of PLD4 mRNA during postnatal development of the mouse cerebellum. A, Semi-quantitative RT-PCR analyses at E18, P0, P3, P7, P12, P15, P21, and P56. Two representative results of PLD4 mRNA expression at cycle number 25 and 28, and GAPDH (control) at cycle 25. B, Relative values (%) of PLD4 GeneChip data are shown in A (peak value at P7 is defined as 100%). GeneChip data shown in Figure 2, which shows peak expression of PLD4 mRNA at P7, is verified by these results. (0.33 MB TIF) [file pone.0013932.s002.tif]

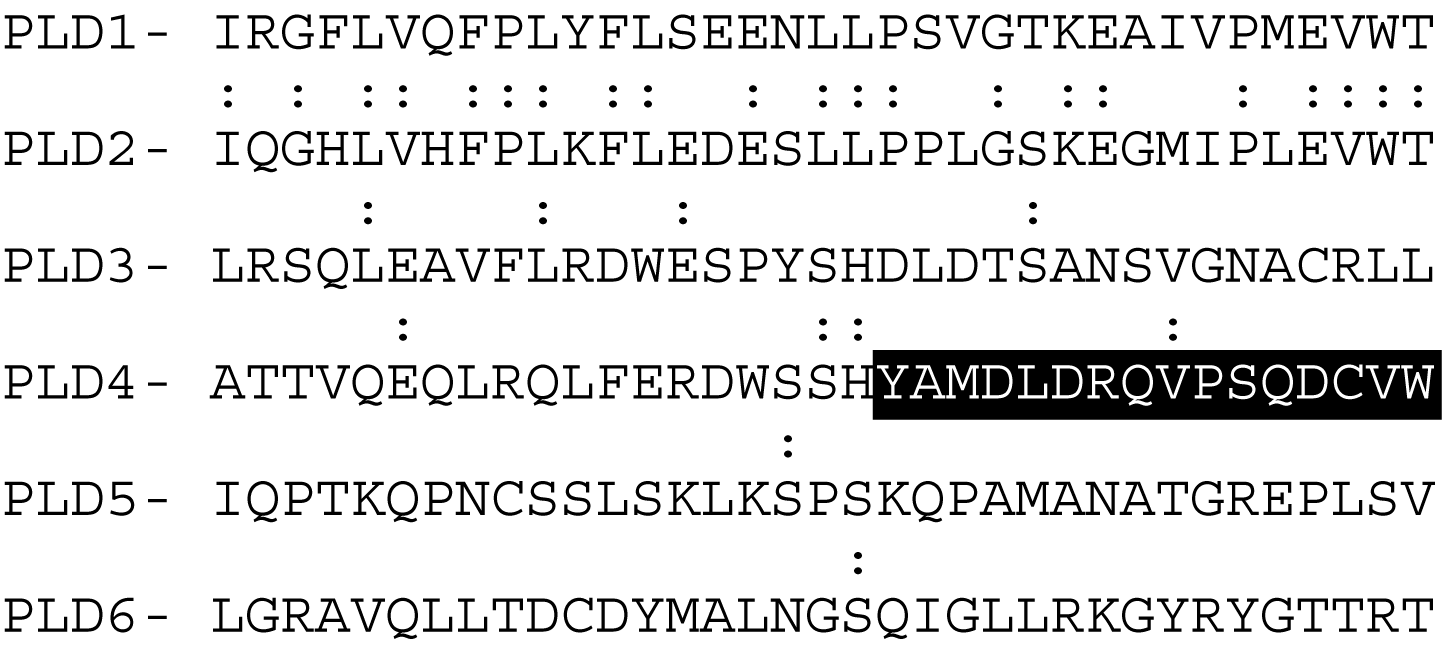

Supplement: Figure S3 — C-terminal amino acid sequences of six mouse PLD family members and the antigen sequence used for generation of the anti-PLD4 antibody. The C-terminal amino acid sequences of six mouse PLD family members (PLD1∼6) are aligned. There is no homology between these C-terminal sequences, except for significant homology between PLD1 and PLD2 (colons indicate identical amino acids). The C-terminal 16 amino acids (highlighted black) of PLD4 were used as the antigenic peptide to generate anti-mouse PLD4 antibody. (0.18 MB TIF) [file pone.0013932.s003.tif]

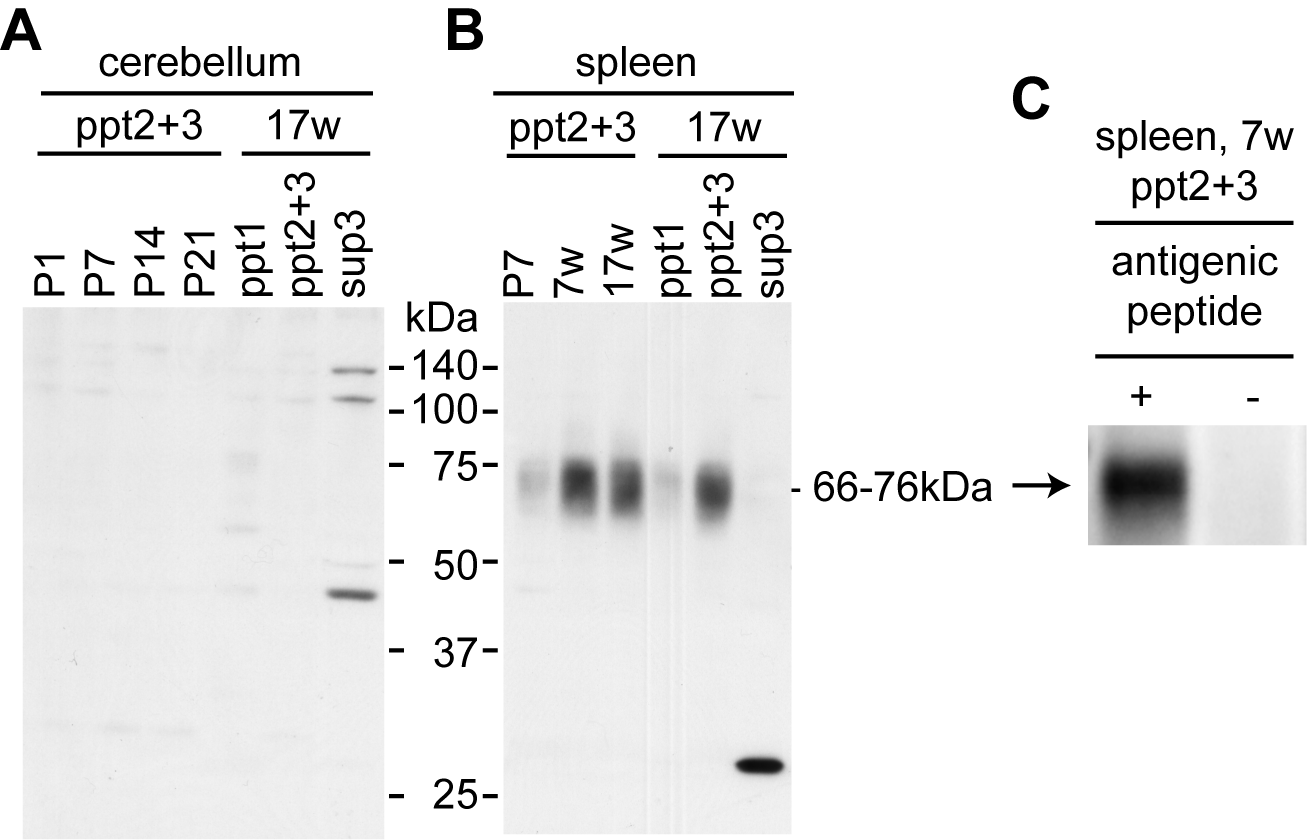

Supplement: Figure S4 — Immunodetection of PLD4 protein by anti-PLD4 antibody. A and B, Subcellular protein fractions (ppt1, ppt2+3, and sup3) prepared from mouse cerebellum (at P1, P7, P14, and P21) and spleen (at P7, 7 week [w], and 17w) were immunoblotted with anti-PLD4 antibody. By employing this conventional method (see Materials and Methods), the antibody specifically reacted with a broad 66∼76-kDa band in the ppt2+3 spleen fraction (B), whereas detected only a trace amount of PLD4 in the cerebellum (A). PLD4 protein levels between spleen and cerebellum are consistent with PLD4 mRNA levels indicated by GeneChip analyses shown in Fig. 3. C, Antigen absorption assay of anti-PLD4 antibody. The ppt2+3 protein fractions prepared from the mouse spleen at 7w were immunoblotted with anti-PLD4 antibody in the presence or absence of antigenic peptide (7 µg/ml) at 4°C overnight. The antigen blocked immunodetection, indicating specificity of the anti-PLD4 antibody to the antigenic amino acid sequence. (0.45 MB TIF) [file pone.0013932.s004.tif]

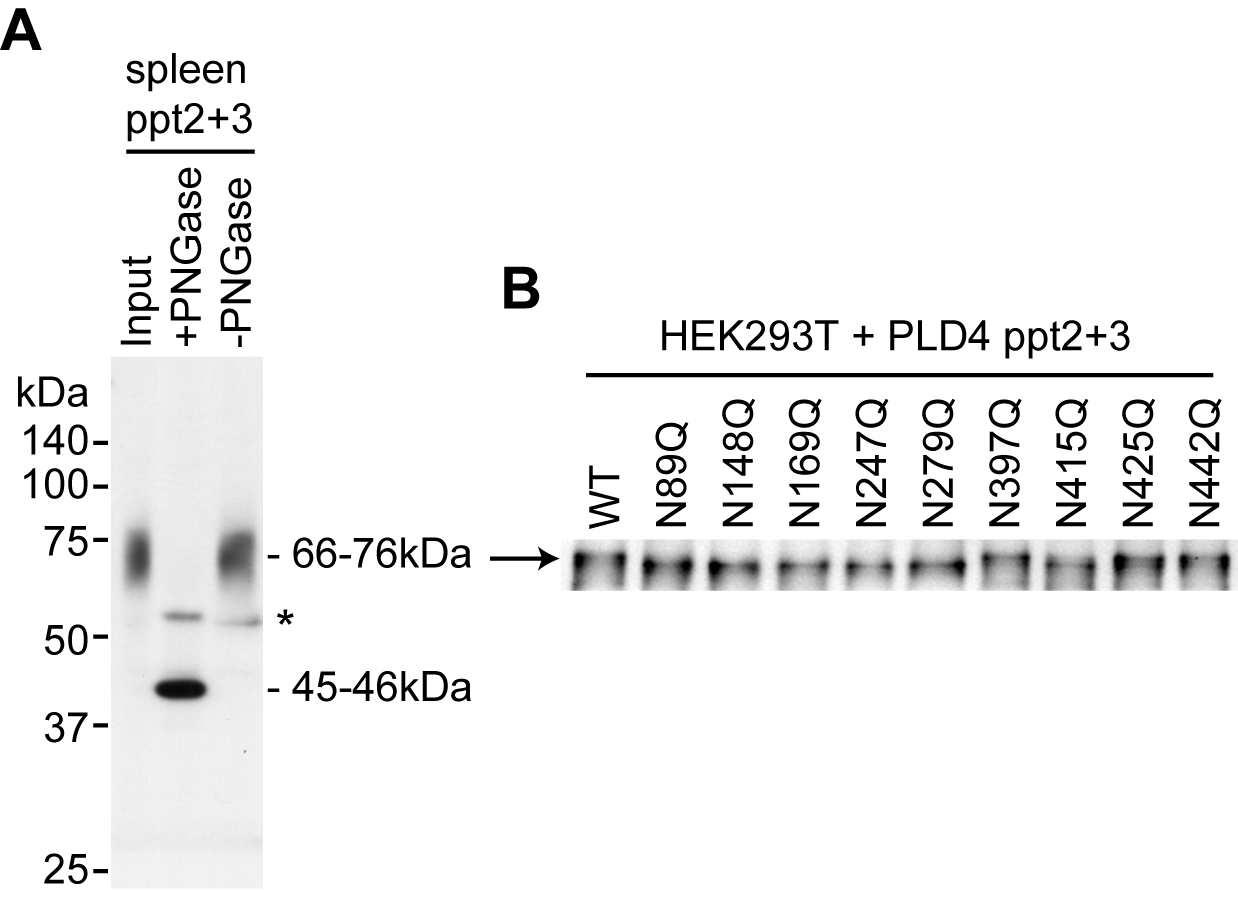

Supplement: Figure S5 — Analyses of glycosylated PLD4 by enzymatic deglycosylation and site-specific mutagenesis. A, Deglycosylation of endogenous PLD4 in the mouse spleen with N-glycosidase-F (PNGase), as shown in Figure 6B. Non-specific bands are indicated by asterisks. B, Site-specific mutagenesis of eight Asn-linked glycan consensus sites in PLD4. Consensus Asn residues (N) at 89, 148, 169, 247, 279, 415, 425, and 442 were individually substituted by Gln (Q). Mutated PLD4, except for N425 and N442Q, exhibits slightly faster mobility than wild-type PLD4 (WT). However, the non-consensus Asn-397 was mutated to Q (N397Q) as a control and exhibits similar mobility as WT. These results suggest that exogenously expressed PLD4 has large and complex glycan moieties and at least six consensus Asn residues at 89, 148, 169, 247, 279, and 415. However, the in vivo glycosylation sites of PLD4 protein remain to be shown. (0.27 MB TIF) [file pone.0013932.s005.tif]
